# Practical Aspects of Risk Assessment in Gastrointestinal Stromal Tumors

**DOI:** 10.1007/s12029-014-9615-x

**Published:** 2014-05-07

**Authors:** R. L. Jones

**Affiliations:** Division of Medical Oncology, Fred Hutchinson Cancer Research Center, University of Washington, 825 Eastlake Avenue East, G-3630, Seattle, WA 98109-1023 USA

**Keywords:** Gastrointestinal stromal tumors, GIST, Risk, Risk assessment, Imatinib, Adjuvant therapy

## Abstract

**Introduction:**

Gastrointestinal stromal tumors (GISTs) are mesenchymal tumors of the gastrointestinal tract, which are characterized in the majority of cases by activating mutations in *KIT* and platelet-derived growth factor receptor alpha (*PDGFRA*). The introduction of tyrosine kinase inhibitors has revolutionized the management of patients with metastatic GIST. However, complete surgical resection remains the mainstay of management for those with localized disease. Recently, three large trials have confirmed the benefit of adjuvant imatinib therapy in patients who were at high risk of recurrence following complete resection. In this setting, it is critical that oncologists understand the various GIST risk assessment criteria and be able to apply these methods to accurately assess the risk of recurrence and the need for adjuvant imatinib therapy.

**Purpose:**

The aim of this review is to outline the risk stratification systems currently available to oncologists who are treating patients with GIST, so they can be optimally applied for clinical decision-making.

## Background

Gastrointestinal stromal tumors (GISTs) are the most common mesenchymal tumors of the gastrointestinal tract. The introduction of the tyrosine kinase inhibitor, imatinib, has revolutionized the management of metastatic GIST. The most common primary sites for such tumors are the stomach (60 %) and small intestine (25–35 %), although they can occur anywhere along the gastrointestinal tract [1, 2]. Resection remains the mainstay of management for patients with localized disease; however, despite adequate resection, there is varying risk of recurrence, ranging from negligible for those with very small GISTs to well over 50 % [2, 3]. Over the last few years, imatinib has shown utility in the adjuvant setting, with the publication/presentation of three randomized trials showing the benefit of adjuvant therapy [4–6]. Consequently, it is clear that accurate and reproducible methods are required to discuss the potential benefits and risks of adjuvant systemic therapy with patients. The majority of patients with low-risk GIST have a favorable outcome after resection and should not receive adjuvant therapy. Over the last decade, several risk stratification systems for resected GIST have been proposed. In addition, others have suggested the use of prognostic nomograms for individualized risk assessment, and mutational status may also have relevance. The aim of this review is to provide a practical guide to the available strategies for risk assessment in GIST.

## Trials of Adjuvant Imatinib

With the publication/presentation of three large randomized trials showing benefit for the administration of adjuvant imatinib, accurate and reproducible methods for risk assessment have gained increasing clinical importance.

The American College of Surgeons Oncology Group (ACOSOG) phase II Z9000 trial assessed the safety and efficacy of 1 year of adjuvant imatinib [6]. This single-arm, open-label, phase II trial enrolled 107 patients with GIST who were at high risk of recurrence following complete resection (tumor size >10 cm, tumor rupture, or <5 peritoneal metastases). With a median follow-up of 7.7 years, 57 of the 106 patients (54 %) had developed recurrent disease and 28 (26 %) had died. Only four patients developed recurrence during the first year. The 1-, 3-, and 5-year recurrence-free survival (RFS) rates were 96, 60, and 40 %, respectively, with a median RFS of 4 years. The 1-, 3-, and 5-year overall survival (OS) rates were 99, 97, and 83 %, respectively, and the median OS has not been reached.

The ACOSOG Z9001 phase III trial randomized 713 patients with tumors ≥3 cm in size to receive either imatinib 400 mg/day or placebo for 1 year after surgery [4]. Accrual to this trial was stopped after the planned interim analysis showed that imatinib significantly improved RFS. At 1 year, RFS was 98 % in the imatinib arm and 83 % in the placebo arm (hazard ratio (HR), 0.35; 95 % confidence interval (CI), 0.22 to 0.53; *p* < 0.0001). Moreover, significant RFS benefit with imatinib was seen in patients at high risk of recurrence (tumor size ≥10 cm) as well as those at intermediate risk (≥6 to <10 cm). With a median follow-up of 19.7 months, no difference in OS was observed between the two arms, but it should be noted that crossover to the imatinib arm was allowed on the development of recurrent disease. Imatinib was well tolerated in this trial. Based on these results, in 2008 the US Food and Drug Administration (FDA) granted accelerated approval of adjuvant imatinib for treatment of KIT-positive GIST, with full approval in 2012. Adjuvant imatinib was also approved in 2009 by the European Medicines Agency (EMA) for KIT-positive GIST with significant risk of relapse.

The fact that many recurrences were observed in this trial after completion of adjuvant imatinib (i.e., approximately 50 % of the tumors ≥10 cm in size recurred during the first 3 years after randomization) suggests that some patients may benefit from a longer duration of therapy. Subsequently, the Scandinavian Sarcoma Group/Arbeitsgemeinschaft Internistische Onkologie trial XVIII (SSG XVIII/AIO) randomized 400 patients with GIST to receive 12 or 36 months of imatinib 400 mg/day after surgical resection of primary tumors [5]. Patients with a high estimated risk of recurrence according to the modified National Institutes of Health (NIH) criteria (i.e., with at least one of the following characteristics: longest tumor diameter >10 cm, mitotic count >10 mitoses per 50 high-power fields (HPF), tumor diameter >5 cm, and mitotic count >5 or tumor rupture) were enrolled in the trial. Patients randomized to 36 months of imatinib had significantly longer RFS compared with those randomized to 12 months of therapy (5-year RFS, 65.6 vs. 47.9 %, respectively; HR, 0.46; 95 % CI, 0.32 to 0.65; *p* < 0.0001). Those who were treated with 36 months of adjuvant imatinib also had significantly longer OS compared with patients who received 12 months of treatment (5-year OS, 92 vs. 82 %; HR, 0.45; 95 % CI, 0.22 to 0.89; *p* = 0.02). More discontinuations were noted in the 36-month group for reasons other than GIST recurrence compared with the 12-month group (51 [25.8 %] vs. 25 patients [12.6 %], respectively). Discontinuation due to adverse events occurred in 13.6 % of patients in the 36-month arm compared with 7.5 % in the 12-month arm.

Consequently, the National Comprehensive Cancer Network (NCCN) and the European Society of Medical Oncology (ESMO) recommend that 36 months of adjuvant imatinib be considered in patients with intermediate- or high-risk tumors [7, 8]. In addition, the FDA and EMA have both updated the label, extending the duration of adjuvant imatinib to 36 months in patients with GIST who are at high risk of recurrence.

Recently, the results of a trial of 900 patients with intermediate- or high-risk resected GIST, who were randomized to receive 2 years of adjuvant imatinib or no adjuvant therapy, have been presented [9]. Patients were stratified by NIH risk criteria, tumor site, and margin status. This trial confirmed the significant benefit of adjuvant imatinib on RFS. The 3-year RFS was 84 and 66 % in the imatinib and the no adjuvant therapy arms, respectively (*p* < 0.001). Similarly, the 5-year RFS was 69 and 63 %, respectively (*p* < 0.001). These investigators have proposed the use of a novel endpoint, imatinib failure-free survival (IFS), defined as the time to when a different tyrosine kinase inhibitor is started. In the high-risk subgroup, a non-statistically significant trend in favor of the adjuvant imatinib was observed in terms of IFS.

The ongoing, non-randomized, single-arm, phase II Post-resection Evaluation of Recurrence-free Survival for gastroIntestinal Stromal Tumors (PERSIST-5) trial is evaluating the efficacy and safety of long-term adjuvant imatinib in patients who are at significant risk for recurrence following complete resection of primary GIST (NCT00867113). Administration of oral imatinib 400 mg/day is planned for up to 5 years or until progression, relapse, or intolerance. The primary endpoint of this trial is RFS.

## Risk Stratification Systems

### NIH Consensus Criteria

The NIH GIST Consensus Criteria were developed by Fletcher et al. [10]. The risk categories proposed by these criteria are shown in Table 1. These criteria utilize two clinical pathological factors, tumor size and mitotic count, allowing recurrence risk to be stratified as very low, low, intermediate, or high. Although the NIH criteria were not derived from actual clinical trial data, subsequent retrospective studies of patients with localized GIST treated with surgery alone have confirmed the prognostic value of both tumor size and mitotic count. A study by the Spanish Group for Sarcoma Research (GEIS) of 162 patients with GIST, treated between 1994 and 2001, found that in a multivariate analysis for RFS, the presence of high cellularity and deletions in codons 557–558 within *KIT* exon 11 were associated with recurrence [11]. Similarly, DeMatteo et al. evaluated 107 patients with localized GIST treated between 2001 and 2003 [12]. Multivariate analysis of factors predictive of recurrence in this and the subsequent phase III adjuvant imatinib trial found that mitotic rate ≥5, tumor size ≥10 cm, and primary tumor location were independent factors. A number of other studies have confirmed the prognostic importance of tumor site [3, 13–15]. Furthermore, the prognostic utility of the NIH criteria has been confirmed in six large cohort studies [16–20]. The NIH criteria clearly are useful and applicable, despite the fact that tumor site is not included as a prognostic factor in this system.

**Table 1:** NIH-Fletcher criteria for GIST risk assessment

Risk category	Primary tumor size (cm)^a^	Mitotic count (per 50 HPF)^b^
Very low risk	<2	<5
Low risk	2–5	<5
Intermediate risk	<5	6–10
	5–10	<5
High risk	>5	>5
	>10	Any mitotic rate
	Any size	>10

Adapted with permission from Fletcher et al. [10]

*GIST* gastrointestinal stromal tumor, *HPF* high-power fields, *NIH* National Institutes of Health

^a^Size is based on single largest dimension

^b^Mitotic index should be standardized based on surface area examined and measured in the most proliferative area of the tumor

### American Forces Institute of Pathology Criteria

The American Forces Institute of Pathology (AFIP) criteria were developed by analyzing a large data set of patients with long-term follow-up [2, 18, 21, 22]. GISTs arising from the stomach have generally better prognosis than those arising from the small bowel or rectum; consequently, the AFIP criteria incorporate tumor site as well as tumor size and mitotic count (Table 2). Tumor size is categorized into four groups: <2 cm, >2 to ≤5 cm, >5 to ≤10 cm, and >10 cm. Mitotic count is classified into two groups: ≤5 or >5 mitoses per 50 HPF. Tumor sites identified in this classification are stomach, duodenum, ileum/jejunum, and rectum.

**Table 2:** AFIP criteria for GIST risk assessment

	Tumor parameter		Patients with progressive disease during follow-up and characterization of malignant potential (%)	
Group	Tumor size (cm)	Mitotic count (per 50 HPF)	Gastric GIST	Small intestinal GIST
1	≤2	≤5	0, none	0, none
2	>2 to ≤5	≤5	1.9, very low	4.3, low
3a	>5 to ≤10	≤5	3.6, low	24, moderate
3b	>10	≤5	12, moderate	52, high
4	≤2	>5	0^a^	50^a^
5	>2 to ≤5	>5	16, moderate	73, high
6a	>5 to ≤10	>5	55, high	85, high
6b	>10	>5	86, high	90, high

Adapted with permission from Miettinen and Lasota [2]

*AFIP* Armed Forces Institute of Pathology, *GIST* gastrointestinal stromal tumor, *HPF* high-power fields

^a^Tumor categories with very small numbers of cases

### Modified NIH Criteria (Joensuu Risk Criteria)

Joensuu has proposed a modified version of the NIH risk assessment system that also includes tumor location and rupture as high-risk factors [23]. This system utilizes four prognostic factors: tumor size, mitotic count, tumor site, and tumor rupture (Table 3). Several studies have documented the high risk of recurrence associated with tumor rupture in GIST [17, 18, 24]. Often tumors that are large and have a non-gastric location tend to rupture, but nonetheless, tumor rupture has independent prognostic information over size, site, and mitotic count [23]. An important point to note is that this system classifies patients with small (≤5 cm), non-gastric GISTs and mitotic counts >5 per 50 HPF and those with non-gastric tumor sizes between 5.1 and 10 cm and <5 mitoses per 50 HPF as having a high risk of recurrence (in contrast to the NIH system).

**Table 3:** Joensuu criteria for GIST risk assessment

Risk category	Tumor size (cm)	Mitotic index (per 50 HPF)	Primary tumor site
Very low	<2	≤5	Any
Low	2.1–5	≤5	Any
Intermediate	2.1–5	>5	Gastric
	<5	6–10	Any
	5.1–10	≤5	Gastric
High	Any	Any	Tumor rupture
	>10	Any	Any
	Any	>10	Any
	>5	>5	Any
	2.1–5	>5	Non-gastric
	5.1–10	≤5	Non-gastric

Adapted with permission from Joensuu [23]

*GIST* gastrointestinal stromal tumor, *HPF* high-power fields

Joensuu et al. performed a pooled analysis of 2,560 patients, who had undergone surgery alone for GIST, from 10 studies comparing the three risk stratification systems described above [14]. They found that large tumor size, high mitotic count, non-gastric primary site, tumor rupture, and male sex were independent adverse prognostic factors. The analysis revealed that all three risk stratification systems were strongly associated with RFS. The authors also observed that most recurrences occurred within the first 5 years after surgery, but occasionally late recurrences were seen. Joensuu et al. also evaluated prognostic contour heat maps using continuous non-linear modeling of tumor size and mitotic count with incorporation of tumor site and rupture. Contour maps were more accurate than the risk stratification systems in predicting risk of recurrence (receiver operating characteristic (ROC) curve analysis: area under the ROC curve, 0.88; 0.86–0.90) [14].

It has been suggested that other tumor characteristics, including infiltration of adjacent structures, serosal invasion, necrosis, high cellularity, and tumor vascularity, could be utilized as risk factors [25]. However, these characteristics are currently not routinely used.

## Prognostic Nomograms

In an attempt to individualize risk of recurrence, Gold et al. [3] and Rossi et al. [24] have proposed two nomograms for risk assessment in patients with resected GIST. Gold et al. developed a nomogram to predict RFS in a data set of 127 patients who were treated at a single institution [3]. The nomogram incorporates size, mitotic index, and tumor site to predict 2- and 5-year RFS. This nomogram has been validated in two external series: GEIS (*N* = 212) and the Mayo Clinic (*N* = 148) data sets. Of note, this nomogram categorizes mitotic count as either ≤5 or >5 mitoses per 50 HPF.

The nomogram proposed by Rossi et al. is also based on tumor size, mitotic count, and tumor site. In contrast to the Gold nomogram, however, tumor size and mitotic index are included as continuous variables [24]. This nomogram was developed through a retrospective analysis of 929 patients treated at 35 Italian centers between 1980 and 2000. It may be used to predict OS; however, this nomogram has not been externally validated. In addition, due to the lack of complete information on recurrence, this nomogram cannot be used to predict RFS.

## Mutational Status

Certain mutations in *KIT* and platelet-derived growth factor receptor alpha (*PDGFRA*) are known to have prognostic or predictive implications. For example, *KIT* exon 9 mutations and exon 11 deletions involving codons 557–558 are associated with poor outcome, and the *PDGFRA* mutation D842V is associated with resistance to imatinib [11, 15, 26–29]. However, conflicting data have been published with regard to mutational status and prognosis in resected localized GIST [11, 15, 18, 30–41]. Ernst et al. first reported that *KIT* mutation was associated with decreased survival (*p* = 0.001) [30], whereas Kim et al. found a correlation between the presence of *KIT* mutation and RFS (overall risk, 5.6) [33]. Similarly, Taniguchi et al. concluded that *KIT* mutation was an independent prognostic factor for OS and disease-specific survival (DFS) [31]. Deletion of codons 557–558 was found to be an independent prognostic factor in multivariate analysis (relative risk, 2.57; 95 % CI, 1.25 to 5.31) [11], which was subsequently confirmed [39]. DeMatteo et al. demonstrated that the absence of point mutation or insertion in *KIT* exon 11 and deletions of codon 557 or 558 in *KIT* exon 11 were significant predictors of recurrence in univariate analysis (*p* ≤ 0.002), but not multivariate analysis. In contrast, Singer et al. had shown earlier that the presence of *KIT* exon 11 deletion or insertion was an adverse independent prognostic factor for DFS (HR, 4; *p* = 0.006) [32]. However, Rutkowski et al. [18], Tzen et al. [35], and Koay et al. [37] reported no correlation between mutational status (presence of *KIT* exon 11 mutation, mutation type) and DFS, risk of recurrence, or patient outcome, respectively. In the analysis performed by Gold et al., incorporation of mutational status did not improve the accuracy of their nomogram [3], but data on mutational status were not available for one of the three data sets used to develop this nomogram [11, 15, 19].

Corless and colleagues analyzed the pathological and molecular features associated with outcome in the ACOSOG Z9001 trial. Three hundred twenty-eight patients were randomized to the placebo arm and 317 to the imatinib arm. Six hundred forty-five tumor specimens were available for mitotic rate or mutational analysis. On multivariate analysis of patients within the placebo arm, tumor size, small bowel location, and mitotic rate were associated with RFS. Mutational status was not associated with RFS [42].

Further analysis in other large series with long-term follow-up will be required to better define any role of mutational status into GIST risk stratification systems.

## Conclusions

Despite their undoubted utility and applicability, all of these methods for GIST risk assessment have limitations. The currently available risk stratification schemes for patients with resectable GIST are relatively straightforward and reliable. The application of these schemes can reduce the overtreatment of patients with fully resected GIST and in patients with low-risk disease that has likely been cured by surgery. However, a major point regarding these systems is the application of cut points for mitotic count and tumor size and whether these are optimal. For instance, the cut points for mitotic count categorization could lead to markedly different estimations of recurrence for patients with a mitotic count close to 5 mitoses per 50 HPF. Furthermore, no reference is made to whether the mitotically most active area of a tumor should be evaluated. The evaluation of mitotic count can be subjective and vary between observers. Furthermore, the number of mitoses detected is dependent on tissue fixation time and different-sized microscopic HPFs [43]. Tumor size determination can also potentially be affected when the specimen is measured in relation to the time of resection until fixation.

Maki has suggested a simple way to stratify the risk for primary GIST, using the “rule of fives” for low- and high-risk disease [44]. Intermediate- to high-risk gastric GISTs are both >5 cm in size and have >5 mitoses per 50 HPF. Furthermore, non-gastric GISTs are high risk if they are either >5 cm or have >5 mitoses per 50 HPF. This simple rule allows all GISTs with a >50 % risk of recurrence to be categorized as well as the intermediate-risk group of 5–10 cm small bowel GISTs with low mitotic rate (AFIP group 3a). However, caution should be emphasized, as this is a much simplified stratification strategy.

Despite their limitations, it is clear that the three widely used risk stratification systems are easily applicable and accurate in predicting risk of recurrence in patients who have undergone surgery for localized GIST. It is likely that risk stratification in patients with localized GIST will continue to evolve over the next few years, with the potential incorporation of mutational status and further data regarding follow-up of patients treated with adjuvant imatinib.
